# A Local Region of Interest Imaging Method for Electrical Impedance Tomography with Internal Electrodes

**DOI:** 10.1155/2013/964918

**Published:** 2013-07-08

**Authors:** Hyeuknam Kwon, Alistair L. McEwan, Tong In Oh, Adnan Farooq, Eung Je Woo, Jin Keun Seo

**Affiliations:** ^1^Department of Computational Science and Engineering, Yonsei University, Seoul 120-749, Republic of Korea; ^2^The School of Electrical and Information Engineering, The University of Sydney, Sydney NSW 2006, Australia; ^3^Impedance Imaging Research Center and Department of Biomedical Engineering, Kyung Hee University, Yongin 446-701, Republic of Korea

## Abstract

Electrical Impedance Tomography (EIT) is a very attractive functional imaging method despite the low sensitivity and resolution. The use of internal electrodes with the conventional reconstruction algorithms was not enough to enhance image resolution and accuracy in the region of interest (ROI). We propose a local ROI imaging method with internal electrodes developed from careful analysis of the sensitivity matrix that is designed to reduce the sensitivity of the voxels outside the local region and optimize the sensitivity of the voxel inside the local region. We perform numerical simulations and physical measurements to demonstrate the localized EIT imaging method. In preliminary results with multiple objects we show the benefits of using an internal electrode and the improved resolution due to the local ROI image reconstruction method. The sensitivity is further increased by allowing the surface electrodes to be unevenly spaced with a higher density of surface electrodes near the ROI. Also, we analyse how much the image quality is improved using several performance parameters for comparison. While these have not yet been studied in depth, it convincingly shows an improvement in local sensitivity in images obtained with an internal electrode in comparison to a standard reconstruction method.

## 1. Introduction

 Electrical Impedance Tomography (EIT) is an attractive electrical properties imaging technique for medical applications due to its speed, safety, relatively low cost, and ability to display unique tissue conductivity information. Conventionally, surface electrodes are used to apply currents and record voltages from the boundary of the object of interest such as the thorax, breast, or head. These measurements are used to reconstruct the internal tissue impedances or conductivities using various image reconstruction methods where sensitivity matrix-based approaches are commonly used. Unfortunately, these measurements are insensitive to local impedance changes away from the measuring positions, whereas they are very sensitive to the boundary geometry and impedance changes near the electrodes. To improve the sensitivity and distinguishability in some local internal regions, the use of internal electrodes has been suggested by several researchers [1, 2]. These previous works were concentrated in cardiac applications of EIT as catheters are routinely introduced into various locations during cardiac monitoring, electrophysiology (EP mapping), or cardiac radiofrequency ablation (RFA).

EIT with internal electrodes could also be applied to monitoring RFA of liver tumors. RFA is widely used for the treatment of liver tumors such as hepatocellular carcinoma (HCC) and metastatic tumors since many patients are not eligible for surgical resection due to advanced disease, unfavorable location, or impaired clinical condition [3]. Numerous studies have reported that RFA is the most minimally invasive treatment for liver tumors and evaluated it as a successful therapeutic modality, providing reliable outcomes even though its posttreatment recurrence rate is higher than that of cryoablation and resection [4]. Ultrasound and magnetic resonance imaging (MRI) commonly provide the guidance of RFA because they allow real-time visualization of probe placement and high contrast images for tumor and normal tissues [5–8]. Contrast-enhanced computed tomography (CT) and MRI are widely used methods to evaluate ablated lesions by comparing the differences of lesions before and after RF ablation. However, none of these are able to continuously monitor the temperature and changes in properties of cancerous tissue and normal tissue during RFA process. 

EIT does have this potential due to its high speed, sensitivity to the conductivity change of tumor and normal tissue at various frequencies [9, 10], and its ability to monitor tissue temperature in real time [11, 12]. In both cardiac EIT and EIT for monitoring liver tumour RFA, there are opportunities to add an additional internal electrode close to the region of interest (ROI) to improve EIT sensitivity to internal tissues and particularly to the tissue within the ROI. In both applications, a local imaging reconstruction method with high sensitivity within the ROI is desired since there are significant effects from conductivity changes outside the ROI such as ventilation, perfusion, movement of the lungs, diaphragm, gastric activity, and blood flow in large vessels [13].

In this paper, we propose a local sensitivity matrix-based imaging method to increase the sensitivity in a local region of interest near the internal electrode and decrease the sensitivity outside the region of interest to progress the application of EIT to cardiac monitoring and liver RFA. To further increase sensitivity to the ROI, we propose a new arrangement of surface electrodes where the electrode spacing is not equal but more near the ROI. In order to evaluate the performance of the local ROI imaging method, we perform numerical simulations and phantom experiments.

## 2. Method

### 2.1. Conventional EIT Reconstruction Method

Let a bounded domain *Ω* represent the subject to be imaged. Surface electrodes *ℰ*_*j*_ for *j* = 1,2,…, *E* are attached to the boundary ∂*Ω*, where *E* is the total number of electrodes. Let *γ*_*t*,*ω*_(**r**) = *σ*_*t*,*ω*_(**r**) + *iωϵ*_*t*,*ω*_(**r**) denote the complex conductivity at time *t*, angular frequency of *ω*, and position **r**. When we inject a sinusoidal current $I\sin(\omega \tilde{t})$ at an angular frequency of *ω* between a chosen pair of electrodes, a voltage distribution $v_{t,\omega }(r)\sin(\omega \tilde{t}+\theta_{t,\omega }(r))$ is formed at the position **r**. Here, *t* is used for expressing a slow-time change in the complex conductivity distribution, and $\tilde{t}$ is used for the fast-time change to represent time-harmonic fields. The induced time-harmonic potential *u*_*t*,*ω*_(**r**) = *v*_*t*,*ω*_(**r**)*e* ^*iθ*_*t*,*ω*_(**r**)^ satisfies the following elliptic partial differential equation [14]:

(1) ∇·(γt,ω(r)∇ut,ω(r))=0, for  r∈Ω,  −γt,ω∇ut,ω·n=g, on⁡  ∂Ω,

where **n** is the outward unit normal vector on ∂*Ω* and *g* is the corresponding Neumann data on ∂*Ω* due to the injection current.

Static imaging in EIT is difficult due to its fundamental limitations in handling boundary geometry and uncertainty in electrode position. Time-difference EIT (tdEIT) and frequency-difference EIT (fdEIT) use time and frequency difference data, respectively, so that the data subtraction can effectively cancel out common errors related to boundary geometry [14–18]. The difference imaging in EIT is based on linear approximations of the following identities:

- time-difference EIT  ∇·((∂/∂*t*)*γ*_*t*,*ω*_(**r**)∇*u*_*t*,*ω*_(**r**)) = −∇·(*γ*_*t*,*ω*_(**r**)∇(∂/∂*t*)*u*_*t*,*ω*_(**r**)),
- frequency-difference EIT  ∇·((∂/∂*ω*)*γ*_*t*,*ω*_(**r**)∇*u*_*t*,*ω*_(**r**)) = −∇·(*γ*_*t*,*ω*_(**r**)∇(∂/∂*ω*)*u*_*t*,*ω*_(**r**)).

Let the angular frequency *ω* be fixed. Let *u*_*t*,*ω*_^*j*^ denote the time-harmonic potential due to *j*th injection current between the adjacent pair of electrodes *ℰ*_*j*_ and *ℰ*_*j*+1_. The boundary voltage between *ℰ*_*k*_ and *ℰ*_*k*+1_ due to the *j*th injection current can be approximated as

(2) Vj,k(t)≈I|ℰ|(∫ℰkut,ωjdS−∫ℰk+1ut,ωjdS)≈∫Ωγt,ω∇ut,ωj·∇ut,ωkdr for  j,k=1,…,E,

where |*ℰ*| is the surface area of the electrode. The last identity in (2) comes from (1) and divergence theorem. We collect *E* ^2^ number of boundary voltage data for a sequence of time *t* = *t*_1_, *t*_2_, *t*_3_,…:

(3) V(t):=(V1,1(t),V1,2(t),…,V1,E(t)︸  1st    current  ,     V2,1(t),…,V2,E(t)︸  2nd    current  ,…,VE,1(t),…,VE,E(t)︸  Eth    current  )T,

where ()^*T*^ is the transpose. Here, any index number must be understood as a modulus of *E*.

The time-difference data due to the time change of *δγ* : = *γ*_*t*_2_,*ω*_ − *γ*_*t*_1_,*ω*_ has the following relation:

(4) $$\begin{array}{c} V_{j,k}\left(t_{2}\right)-V_{j,k}\left(t_{1}\right)=-\int_{\Omega }\underset{\delta \gamma }{\underset{︸}{\left(\gamma_{t_{2},\omega }-\gamma_{t_{1},\omega }\right)}}\nabla u_{t_{2},\omega }^{j}\cdot \nabla u_{t_{1},\omega }^{j}dr. \end{array}$$

The linearized method is based on the following rough approximation:

(5) $$\begin{array}{c} \delta V:=V_{j,k}\left(t_{2}\right)-V_{j,k}\left(t_{1}\right)\approx \int_{\Omega }\delta \gamma \nabla u_{*}^{j}\cdot \nabla u_{*}^{k}dr, \end{array}$$

where *u*_∗_^*j*^ is the potential of (1) corresponding to a reference conductivity *γ* = *γ*_∗_.

Discretizing the domain of interest into pixels and assuming that *δγ* is constant on each pixel *q*_*n*_, the time-difference EIT problem of (5) can be changed to solve the following linear system:

(6) $$\begin{array}{c} 𝕊\delta \gamma \;=\delta V. \end{array}$$

The *n*th column of the sensitivity matrix *𝕊* is

(7) sn=(∫qn∇u∗1·∇u∗1dr,…,∫qn∇u∗j·∇u∗kdr,   …,∫qn∇u∗E·∇u∗Edr)T.

Hence, the EIT problem is to find a best linear combination of column vectors **s**_1_,…, **s**_*N*_ which produces *δ* **V**:

(8) $$\begin{array}{c} \delta V\approx \delta \gamma_{1}s_{1}+\cdots +\delta \gamma_{N}s_{N}. \end{array}$$

The column vector **s**_*k*_ of *𝕊* represents sensitivity of current-voltage data at the fixed pixel *p*_*k*_, whereas row vectors of *𝕊* represent sensitivity distribution for a fixed current-voltage data. We refer to this approach (combined with a regularization) to reconstruct images as the conventional method throughout this paper.

### 2.2. Local-ROI Imaging Method for EIT

The local-ROI imaging method for EIT is to provide the image of the conductivity change in a local region of interest (ROI) instead of the image in the entire domain *Ω*. Let a domain *D* be the local region of interest to be imaged. Imagine that the measured data *δ* **V** in (5) is divided into two parts

(9) $$\begin{array}{c} \delta V=\delta V_{D}+\delta V_{\Omega \setminus D}, \end{array}$$

where *δ* **V**_*D*_ is the voltage change in response to the conductivity perturbation *δγ* in the local ROI *D* and *δ* **V**_*Ω*∖*D*_ is the voltage change in response to *δγ* in *Ω*∖*D*. With proper arrangement, we may assume that the first *R* column vectors of *𝕊* are sensitivity vectors to pixels in ROI *D* and the other column vectors are sensitivity vectors to pixels in *Ω*∖*D*. Then the sensitivity matrix *𝕊* can be decomposed into

(10) $$\begin{array}{c} 𝕊=\left[\begin{array}{cc} {𝕊}_{D} & O \end{array}\right]+\left[\begin{array}{cc} O & {𝕊}_{\Omega \setminus D} \end{array}\right], \end{array}$$

where **O** represents a proper size of zero matrix,

(11) $$\begin{array}{c} {𝕊}_{D}=\left[\begin{array}{ccc} \;|\; & & \;|\;\\ s_{1} & \cdots & s_{R}\\ \;|\; & & \;|\; \end{array}\right],\;\;{𝕊}_{\Omega \setminus D}=\left[\begin{array}{ccc} \;|\; & & \;|\;\\ s_{R+1} & \cdots & s_{N}\\ \;|\; & & \;|\; \end{array}\right]. \end{array}$$

**Figure 1:**
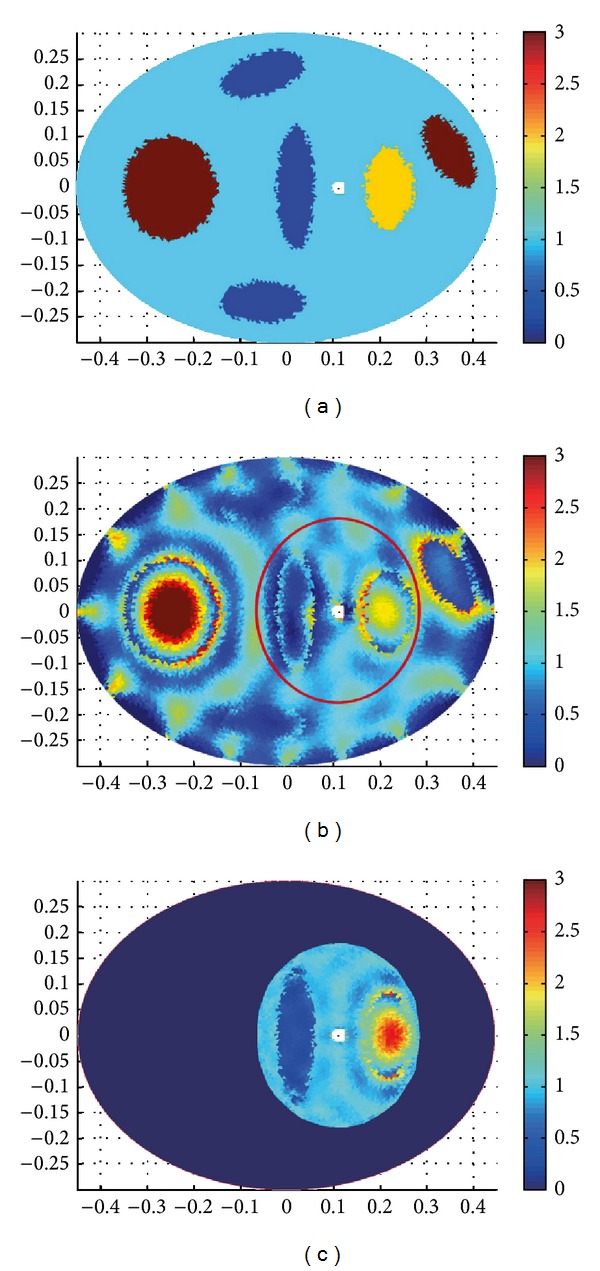
Reconstructed images using *δ* **V** and *δ* **V**_*D*_ via numerical simulations: (a) True image *δ* **γ**, (b) reconstructed image using *δ* **V**, and (c) reconstructed image using *δ* **V**_*D*_.

If we could extract the data *δ* **V**_*D*_ by filtering out *δ* **V**_*Ω*∖*D*_, the global problem (6) can be changed into the local problem:

(12) $$\begin{array}{c} {{𝕊}_{D}\delta \gamma }_{D}=\delta V_{D}, \end{array}$$

where *δ* **γ**_*D*_ = [*δγ*_1_, *δγ*_2_,…, *δγ*_*R*_]^*T*^.  Figure 1(c) shows the reconstructed image of *δ* **γ**_*D*_ using the localized linear system (12) via numerical simulation. Comparing this local image with the standard EIT image reconstruction shown in Figure 1(b), it would be desirable to filter out *δ* **V**_*Ω*∖*D*_ to enhance image resolution. 

For the local imaging in the ROI *D*, we aim to develop a method of extracting **V**_*D*_ from the full data **V**. In order to eliminate the unrelated data **V**_*Ω*∖*D*_ in the linear system (6), we need to find an optimal matrix Φ such that

(13) $$\begin{array}{c} \Phi =\arg\;\underset{\Phi }{\min}||\Phi^{T}\delta V-\Phi^{T}\delta V_{D}||. \end{array}$$

Here, arg min⁡_Φ_ *η*(Φ) gives a matrix Φ at which *η*(Φ) is minimized. If Φ satisfies ||Φ^*T*^ *δ* **V** − Φ^*T*^ *δ* **V**_*D*_|| ≈ 0, then it eliminates the unrelated data **V**_*Ω*∖*D*_ and we get the localized linear system corresponding to (12):

(14) $$\begin{array}{c} \Phi^{T}𝕊\delta \gamma \approx \Phi^{T}\delta V_{D}\;\;\left(\text{since}\;\;\Phi^{T}\delta V\approx \Phi^{T}\delta V_{D}\right). \end{array}$$

**Figure 2:**
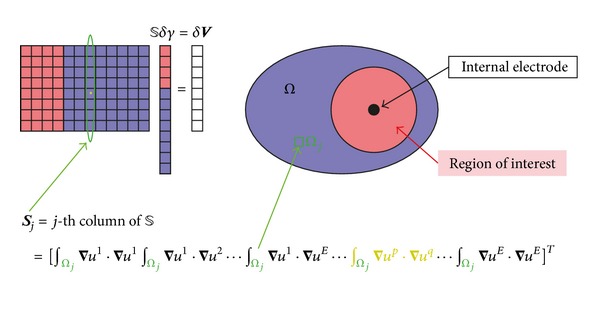
Column vectors of the sensitivity matrix are related with pixels in *Ω*.

Note that the quantity *λ*_*D*_ : = min⁡_Φ_||Φ^*T*^ *δ* **V** − Φ^*T*^ *δ* **V**_*D*_|| depends on the electrode configuration and mesh structure that determines the structure of column vectors of *𝕊* as shown in Figure 2. The *λ*_*D*_ may not be small when the sensitivity matrix *𝕊* is highly ill-conditioned. If the column vectors **s**_1_,…, **s**_*R*_ are orthogonal to **s**_*R*+1_,…, **s**_*N*_, then *λ*_*D*_ = 0 by choosing Φ whose rows consist of the column vectors **s**_1_,…, **s**_*R*_. But, this is not possible with the standard EIT electrode configuration. We try to find an optimal Φ which minimizes *λ*_*D*_. Indeed, cross-correlation *μ*(*j*) of column vector in *𝕊* is big if **s**_*j*_ is correlated with column vectors in ROI; cross-correlation is defined by

(15) $$\begin{array}{c} \mu \left(j\right)=\underset{{\;}_{p_{i}\in \Omega \setminus D}}{\text{avg}}\frac{\left|s_{i}^{T}s_{j}\right|}{{||s_{i}||}_{2}{||s_{j}||}_{2}},\;\text{for}\;\;p_{j}\in D. \end{array}$$

**Figure 3:**
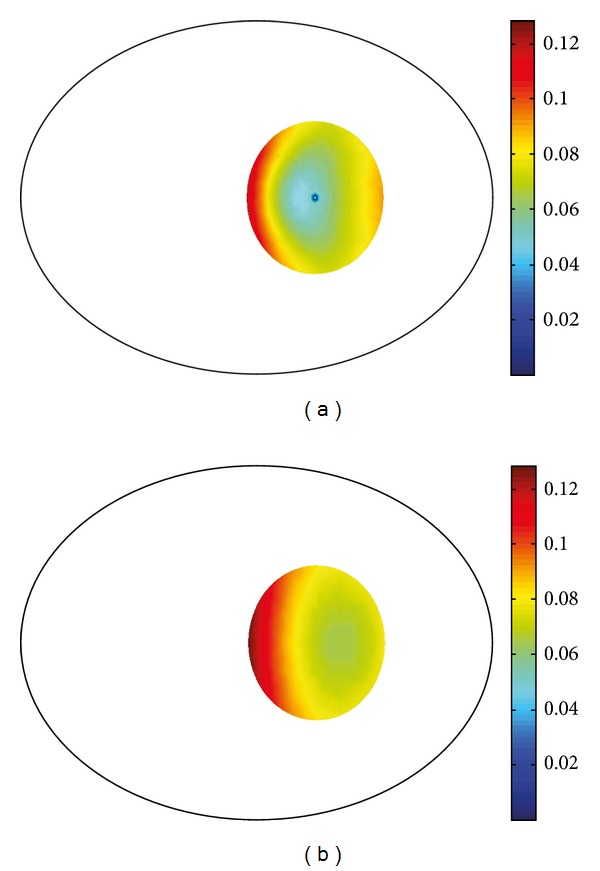
The cross-correlation distribution on *D*: (a) *μ* with internal electrode and (b) *μ* without internal electrode.

Figure 3 shows that cross-correlation values decrease by placing internal electrode.

For finding proper Φ, we propose the following minimization

(16) $$\begin{array}{c} \phi_{k}=\arg\;\underset{\;\;\;\phi \;}{\min}\left(\sum_{p_{j}\notin D}{\left|s_{j}\cdot \phi \right|}^{2}+\alpha {||\phi \;-s_{k}||}_{2}^{2}\right),\;p_{k}\in D, \end{array}$$

where *α* is a suitable parameter. We should note that each **ϕ**_*k*_ is designed to be close and to be parallel to **s**_*k*_ while orthogonal to **s**_*j*_ for each *p*_*j*_ ∉ *D*. The first term in (16), ∑_*p*_*j*_∉*D*_|**s**_*j*_·**ϕ**|^2^, is small when **ϕ** is orthogonal to {**s**_*j*_}_*p*_*j*_∉*D*_. The second term in (16), ||**ϕ**  −**s**_*k*_||_2_^2^, is small if **ϕ** is parallel to **s**_*k*_. We define a matrix Φ whose columns are consisted of {**ϕ**_*k*_}_*p*_*k*_∈*D*_:

(17) $$\begin{array}{c} \Phi =\left(\phi_{1},\;\phi_{2},\ldots ,\phi_{R}\right),\;\text{where}\;\;\overset{R}{\underset{n=1}{⋃}}p_{n}=D. \end{array}$$

We multiply Φ^*T*^ to the linearized system (6):

(18) $$\begin{array}{c} \Phi^{T}𝕊\delta \gamma =\Phi^{T}\delta V. \end{array}$$

Now, we have the linear system (18) with the modified sensitivity matrix Φ^*T*^ *𝕊* with the modified data Φ^*T*^ *δ* **V**. Here, Φ^*T*^ *δ* **V** is regarded as a rough approximation of Φ^*T*^ *δ* **V**_*D*_.

## 3. Numerical Simulations

**Figure 4:**
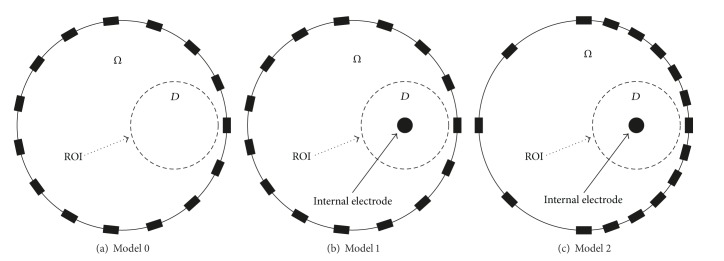
(a) Standard EIT phantom without using internal electrode, (b) equidistant surface electrodes with an internal electrode, and (c) unequally spaced surface electrodes with denser spacing near by the ROI with an internal electrode.

In order to analyse the boundary electrode position and the benefit of using an internal electrode with the proposed local-ROI imaging method, we prepared three different kinds of electrode configuration and applied the conventional and local-ROI imaging methods explained in the previous sections.  Figure 4 shows the cylindrical phantoms with three different electrode configurations. Data obtained from all three electrode configurations were processed by the conventional TSVD reconstruction method. The local-ROI imaging method uses an internal electrode so it was only applied to data obtained with the two internal electrode configurations (Figures 4(b) and 4(c), (Models 1 and 2)). We carry out a total of five numerical simulations, the standard method on Model 0, 1, 2 and the local-ROI method on models 1, 2.

**Figure 5:**
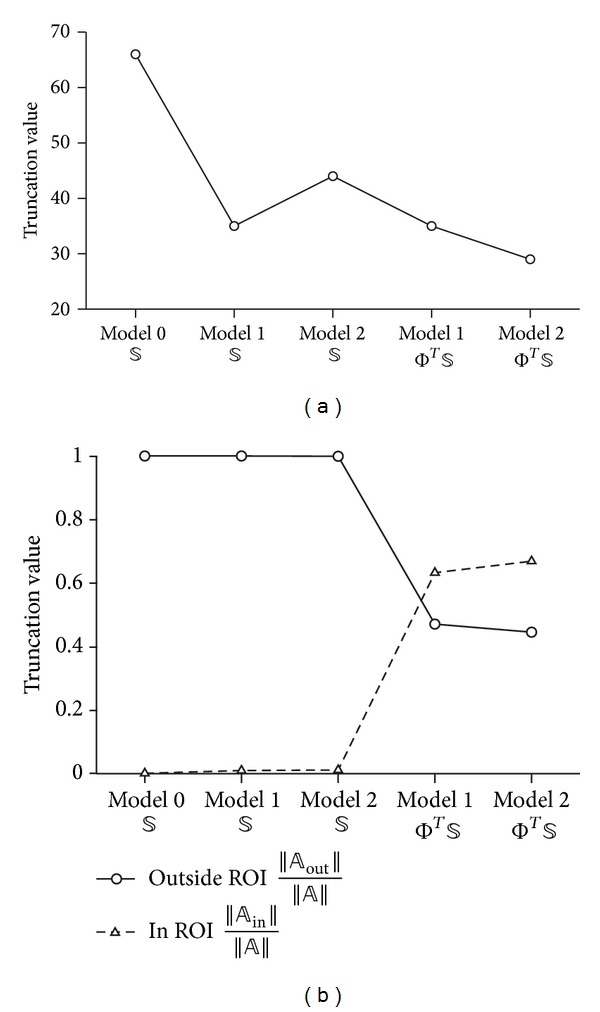
Comparison of the conventional and local-ROI imaging method by (a) singular values required to the same conductivity contrast in images and (b) ratio of norms for elements associated within the ROI and outside the ROI for each case.

To compare sensitivity and robustness to noise in the suggested five cases, we simulated an object of 2 S/m conductivity with 0.1428 diameter at (0.8, 0) in the ROI of 1 S/m saline tank. The radius of the ROI is defined by 5/6 of distance between internal electrode and closest boundary electrode. We used two performance indexes to assess the improvement when using an internal electrode and the proposed linear system (18). First, we computed the singular value threshold to produce the same conductivity contrast of image as shown in Figure 5(a). We repeatedly reconstruct images, updating the singular value threshold, until the same conductivity contrast is produced between the anomaly and background in the reconstructed image. Then we compared the singular value thresholds as a lower number of threshold is an indication of better noise robustness. When we used an internal electrode, the truncated singular value threshold was lower than that without using the internal electrode. Also, the performance of local-ROI imaging method was improved when it was used with unequally spaced surface electrodes with denser spacing near by the ROI. Second, we are concerned about the effect of high contrast anomalies outside the ROI. To investigate this, we examined the sensitivity values of *𝕊* and Φ^*T*^ *𝕊* for each simulation case. The normalized sensitivity values of *𝕊* and Φ^*T*^ *𝕊* from within the ROI and out of ROI are shown separately in Figure 5(b). There was an improved relative sensitivity to the ROI region with the proposed method Φ^*T*^ *𝕊*. To show sensitivity values, we calculated the ratio of the matrix norm ||*𝔸*_in_||/||*𝔸*|| and ||*𝔸*_out_||/||*𝔸*|| for inside ROI and outside ROI respectively, where

  
*𝔸* ∈ {*𝕊* of  Model  0, 1, 2, Φ^*T*^ *𝕊*  of  Model  1, 2},
  
*𝔸*_in_  is  a  submatrix  of  *𝔸*  corresponding  to  ROI,
  
*𝔸*_out_  is  a  submatrix  of  *𝔸* corresponding  to  outside  ROI,
  ||*𝔸*|| : = sup⁡_||**x**||=1_||*𝔸* **x**||.

Note that a matrix norm ||*𝔸*|| shows how much *𝔸* deforms **x**. So, ||*𝔸*_in_||/||*𝔸*|| and ||*𝔸*_out_||/||*𝔸*|| show maximum influence of submatrices *𝔸*_in_ and *𝔸*_out_ for multiplication of *𝔸*.

**Figure 6:**
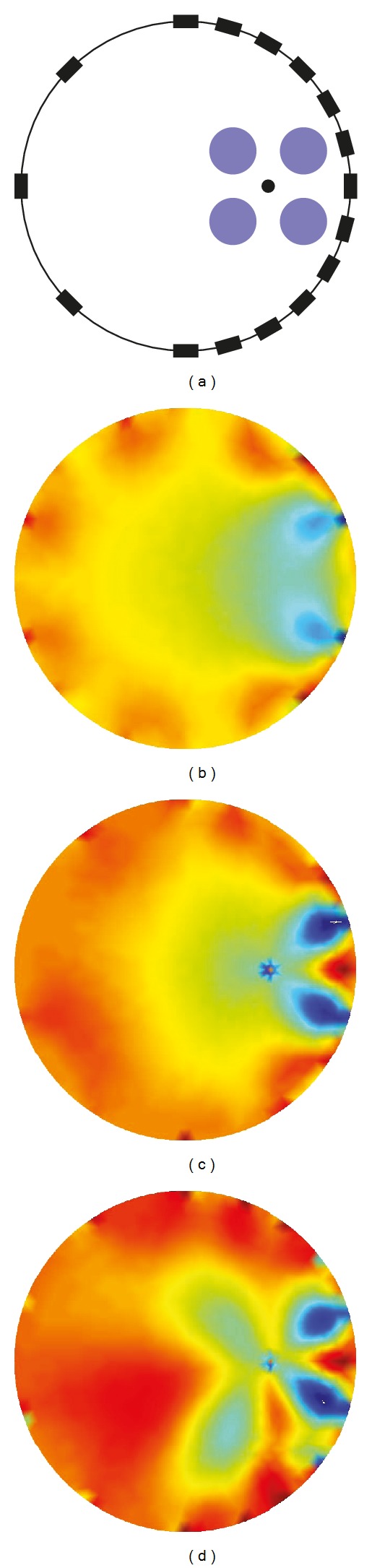
Reconstruction results using simulation data. (a) Simulation configuration for imaging test, (b) conventional TSVD reconstructed image using diagonal injection, (c) TSVD reconstructed image using (6) with an internal electrode, and (d) TSVD reconstructed image using local-ROI imaging method (12) with an internal electrode.

In order to compare the performance of the imaging methods in the reconstructed images, we placed multiple objects which had the same conductivity of 0.029 S/m with 0.01 diameter at (0.02, 0.015), (0.02, −0.015), (0.05, −0.015), and (0.05, −0.015) within the ROI of the 0.0418 S/m saline tank as shown in Figure 6(a). The conventional reconstructed image without an internal electrode was produced by the TSVD algorithm in Model 0 with diagonal current injection (Figure 6(b)). For comparison of the imaging methods using an internal electrode, we positioned an internal electrode at (0.035, 0) in Model 2. Figures 6(b) and 6(c) show images using the conventional TSVD reconstruction algorithm and the local-ROI imaging method. The proposed method with an internal electrode has better sensitivity and detectability in the ROI.

## 4. Experimental Results

To evaluate the performance of the imaging methods in a physical model, we prepared a cylindrical saline tank with the same geometry, ROI, and electrode positions as Figures 4(a) and 4(c) since the denser clustering of boundary electrodes near the ROI (Figure 4(c)) produced better results than the conventional equally spaced electrodes (Figure 4(b)) in the simulation study. The diameter and height of saline tank were 14 cm and 6 cm, respectively. The boundary and internal electrodes were located 3 cm from the bottom in the *z* direction. The *x* and *y* positions of the internal electrode were (3.5, 0) cm relative to the origin (0, 0) at the center of cylindrical tank. The diameter of internal electrode was 0.25 cm and it was covered with insulated rubber except for the end piece of exposed metal. The conductivity of saline was 0.042 S/m. All data was measured by the KHU Mark2.5 EIT system operated at 10 kHz [19].

**Figure 7:**
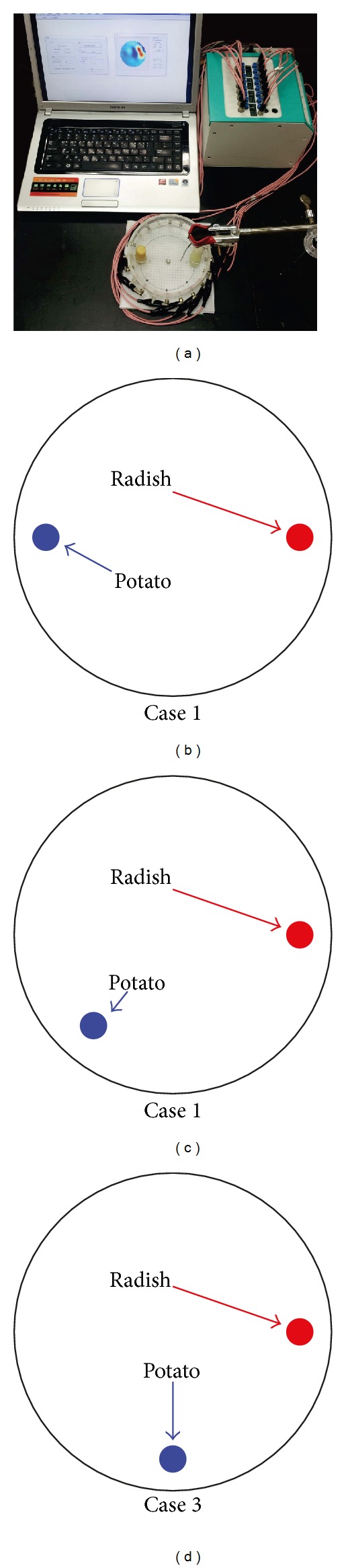
(a) Phantom experimental setup, (b) radish and potato position of case 1, (c) radish and potato position of case 2, and (d) radish and potato position of case 3.

**Figure 8:**
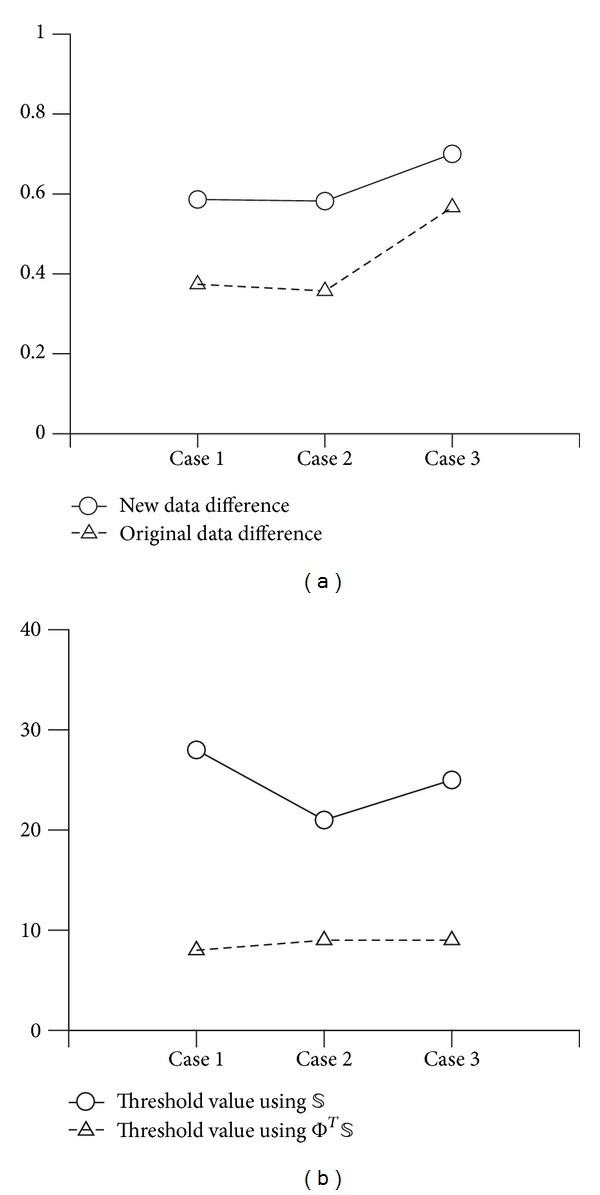
Norm values of difference in experimental data caused by an anomaly outside the ROI in the three different positions given in Figure 7.

We evaluated the imaging methods using two different situations. First, we located a piece of radish inside of ROI (case 0, 1, 2, 3 (5.6, 0)) and a piece of potato outside of ROI (case 1 (−5.6, 0), case 2 (−3.5, −4), case 3 (0, −5.6)) to assess how the sensitivity of each method is influenced by a high conductivity object in the surrounding area of ROI. The diameter and height of both objects were the same at 1.2 cm and 7 cm as shown in Figure 7. The conductivity of radish (0.038 S/m at 10 kHz) was 9.5% lower than saline and the potato (0.029 S/m at 10 kHz) was a higher contrast than the radish with 31% lower conductivity than saline. All data was obtained in the new configuration with an internal electrode and unequal surface electrode spacing as Model 2 in Figure 4(c). All measured data was processed by the conventional TSVD method and the local-ROI imaging method for comparison. We present the norm values of the difference of *δ* **V** and Φ^*T*^ *δ* **V** compared to those values of reference case 0 because it is difficult to analyze images directly from the artifacts due to the large anomaly outside the ROI. Here, we processed reference value by conventional method in case 0 which included only one anomaly (radish) in the ROI.  Figure 8(a) shows the effect of the high conductivity contrasted object located outside of ROI. There is less effect with the new local-ROI imaging method when the object outside the ROI is in the opposite hemicircle to the ROI. As the object approaches the ROI (case 1 to case 3) both methods perform similarly.

Figure 8(b) shows the singular value threshold required to produce the same conductivity contrast for each case. The local-ROI imaging method had similar threshold values for all cases with less than 1.73% variation. However the conventional TSVD method showed larger singular values and large dependence on the high contrast anomaly position.

Secondly, we evaluated the methods from the reconstructed images in the same configuration as the numerical simulation. We put four carrot objects with 0.04 S/m conductivity at 10 kHz in the 0.06 S/m saline tank. The position of each object was (1.5, 2), (5.5, 2), (5.5, −2), and (1.5, −2) cm, respectively. All objects had the same diameter of 2 cm.

**Figure 9:**
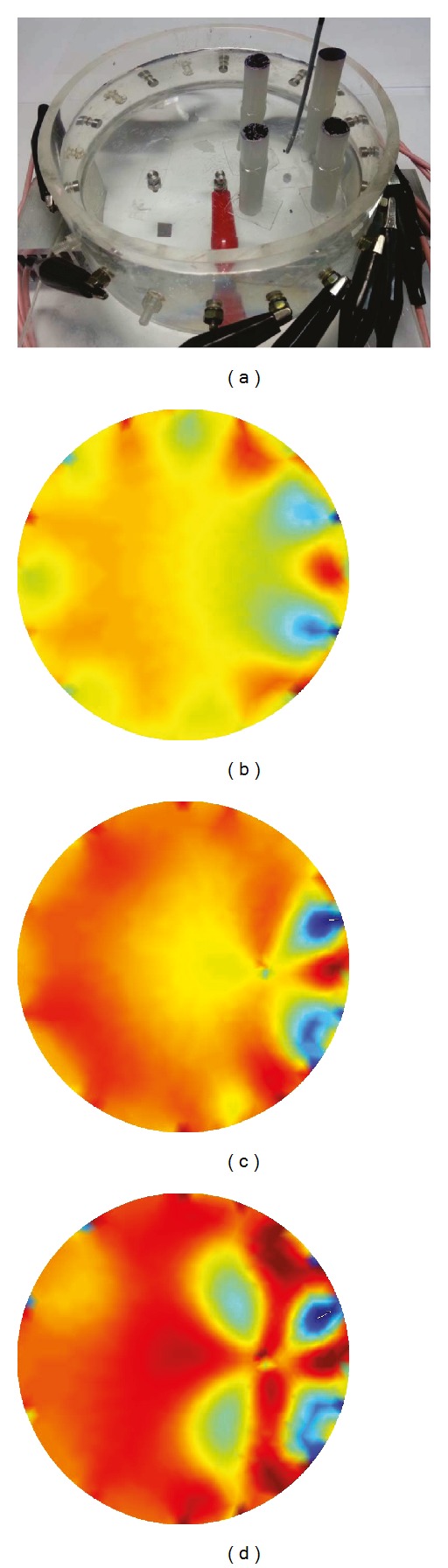
Reconstruction results using experimental data. (a) Experiment configuration for imaging test, (b) conventional TSVD reconstructed image using diagonal injection, (c) TSVD reconstructed image using (6) with an internal electrode, and (d) TSVD reconstructed image using local-ROI imaging method (12) with an internal electrode.

We obtained an image in configuration Model 0 during diagonal current injection and TSVD reconstruction. For algorithm comparison, we measured data in Model 2 and applied both methods separately. Figures 9(a)–9(c) show the reconstructed images. The proposed local-ROI imaging method with an internal electrode can distinguish inner objects better than conventional methods.

## 5. Conclusions and Discussions

 The new local-ROI imaging method has been shown to improve the sensitivity in the ROI region and robustness to noise by comparison of sensitivity matrix values. It provided the approximated linear system with optimized sensitivity matrix to emphasize the detection in ROI. The ROI region is close to an internal electrode and surface electrodes were unequally spaced being more dense close to the ROI. The new method and setup is also less affected by the high conductivity contrasted object outside of ROI. These objects are mimicking a large vessel and several regions of hepatic and metastatic cancers treated by RFA, other gastric organs, or the lungs in cardiac monitoring.

Some performance indexes proposed in this paper described that the sensitivity and detectability were obviously improved in simulation and experiment results. However, the reconstructed images did not show improvements as dramatic as we initially expected. One of the major reasons was the ill-posedness of imaging problem. When we designed the optimal Φ^*T*^ matrix that satisfied condition (16), it could not eliminate the effect of unrelated data **V**_*Ω*∖*D*_ completely. Also, the quality of the experimental reconstructed image was highly dependent on the position of an internal electrode and boundary electrodes because we placed the electrodes with denser spacing near by the ROI. Encouragingly with the pilot results of the local-ROI imaging method using an internal electrode, it shows the feasibility and suggests a new approach to improve the resolution of internal local region. 

An additional improvement which was introduced in this paper was the nonequidistant spacing of electrodes, with the electrodes more densely spaced near the ROI in Model 2 (Figure 4(c)). This showed improved results in simulation and so was used in the experimental setup; however, this may be more sensitive to electrode positions, the location of the ROI, or noise. Interestingly in Figure 5(a) we found that this setup of non-equidistant electrodes (Model 2) showed less sensitivity with the conventional EIT method. This may reflect the preference for symmetry with EIT performed in a circular object. The single ROI we investigated was off centre and once we use our local-ROI focusing algorithm there is an improvement found in the non-equidistant electrode setup (model 2).

The application of EIT to RF ablation monitoring has high potential; however, we need to study more how to separate the conductivity variations which arise simultaneously from temperature changes and tissue property changes. While the temperature coefficient of conductivity in electrolytes of 2% per degree is well known for electrolytes, tissues also exhibit a conductivity dependence on temperature-induced fluid volume shifts which is of the same order of magnitude [20].

Lower frequency measurements could be used to discriminate ablated and nonablated tumor and normal tissues. Liver tumour tissue has a higher conductivity than normal liver tissue over 10 Hz to 1 MHz shown by four terminal impedance measurements in excised tissue. Following an ablation, both tissue types showed significantly increased conductivity over the same frequency range indicating that electrical impedance may be used to differentiate tumor tissue diagnostically, for ablation planning and postablation assessment [10].

The suggested method may be applied in 3D domain without any additional changes to the algorithm. Here we used a 2D domain analysis because applying the suggested method requires a lot of computations on the column vectors of sensitivity matrix corresponding to pixels in ROI [21]. We need to optimize the local-ROI imaging algorithm by focusing on the ROI and investigate ways to increase the coverage area without moving the electrode position. We may combine the frequency-difference EIT [22, 23] with the local-ROI imaging method to apply frequency-difference data.
